# Knowledge, attitude, and practice of patients receiving maintenance hemodialysis regarding hemodialysis and its complications: a single-center, cross-sectional study in Nanjing

**DOI:** 10.1186/s12882-023-03320-0

**Published:** 2023-09-20

**Authors:** Fangfang Xu, Bing Zhuang, Zhongxia Wang, Hao Wu, Xin Hui, Hongyan Peng, Xueqin Bian, Hong Ye

**Affiliations:** Center for Kidney Disease, The Second Affiliated Hospital of Nanjing Medical University, Nanjing, 210000 China

**Keywords:** Uremia, End-stage renal disease, Hemodialysis, surveys and questionnaires, Health Knowledge, Attitudes, Practice

## Abstract

**Background:**

Good knowledge of and attitudes toward hemodialysis and its complications might be expected to promote good practices and improve adherence. This study investigated, the knowledge, attitude, and practice of patients receiving hemodialysis regarding hemodialysis and its complications.

**Methods:**

This cross-sectional study enrolled patients with uremia who were receiving hemodialysis at the Second Affiliated Hospital of Nanjing Medical University (China) between January 9, 2023, and January 16, 2023. A questionnaire was designed that included the following dimensions: demographic/clinical information, knowledge, attitude, and practice. Correlations between knowledge, attitude, and practice scores were evaluated by Pearson correlation analysis.

**Results:**

The analysis included 493 patients (305 males, 61.87%). The average knowledge, attitude, and practice score was 19.33 ± 7.07 (possible range, 0–31), 28.77 ± 3.58 (possible range, 8–40), and 43.57 ± 6.53 (possible range, 11–55) points, respectively. A higher knowledge score was associated with younger age (*P* < 0.001), a higher education level (*P* < 0.001), and not living alone (*P* < 0.001), while a higher practice score was associated with a shorter history of hemodialysis (*P* < 0.001). There were positive correlations between the knowledge and practice scores (*r* = 0.220, *P* < 0.001) and between the attitude and practice scores (*r* = 0.453, *P* < 0.001), although the knowledge and attitude scores were not significantly correlated.

**Conclusions:**

The results provide important insights into the knowledge, attitudes, and practices of patients with uremia in Nanjing (China) regarding hemodialysis and its complications. These findings may facilitate education programs to improve self-care practices in patients receiving maintenance hemodialysis in Nanjing (China).

**Supplementary Information:**

The online version contains supplementary material available at 10.1186/s12882-023-03320-0.

## Background

End-stage kidney disease (ESKD) is a reduction in renal function for more than 3 months, resulting in an estimated glomerular filtration rate of less than 15 mL/min/1.73 m^2^ and/or symptomatic uremia that requires renal replacement therapy [1, 2]. The main causes of ESKD are diabetes mellitus and hypertension, accounting for around three-quarters of cases [3]. The incidence of ESKD has risen substantially in East and Southeast Asia due in part to population aging and poorer health behaviors (i.e., lifestyle and dietary changes) [4]. In 2018, the incidence and prevalence of ESKD in the USA were 390 per million and 242 per million, respectively, and ESKD was more common in African American and Hispanic people than in Caucasian people [5]. A recent study in China estimated the age-and sex-standardized prevalence of kidney disease treated with dialysis to be 419 per million in 2017 [6], while a previous report determined that the annual mortality rate from ESKD was 6.4% [7]. Thus, ESKD remains a major public health issue in China, as elsewhere in the world.

The most prevalent method of renal replacement therapy is hemodialysis [2]. However, hemodialysis is associated with short-term and long-term complications such as intradialytic hypotension, muscle cramps, headache, nausea and vomiting, itching, dialysis disequilibrium syndrome, dialyzer reactions, acute hemolysis, air embolism, bloodstream infections, vascular access stenosis, and development of a catheter-related fibroepithelial sheath [8–10]. Furthermore, adherence to hemodialysis therapy among patients with ESKD is poor [11–13], and it is well established that poor adherence to hemodialysis is associated with poorer outcomes [14]. Factors associated with poor adherence to hemodialysis sessions include not being married, considering it unimportant to follow the dialysis schedule, unavailability of personal transportation, not having a busy lifestyle, not receiving advice from medical professionals regarding the importance of not missing dialysis sessions and less frequent advice from medical professionals about adhering to dialysis [12].

Adequate knowledge regarding hemodialysis and its complications and positive attitudes toward hemodialysis and the prevention of complications would be expected to improve adherence to therapy and, hence, outcomes. Identifying the barriers that reduce adherence to hemodialysis therapy is important because such data can facilitate the design and implementation of interventions to improve adherence. Knowledge, attitude, and practice (KAP) surveys provide useful information regarding the baseline knowledge, attitudes, beliefs, misconceptions, and behaviors towards a health-related topic [15]. Furthermore, the data obtained by KAP surveys can help healthcare professionals develop and implement education programs to overcome issues and barriers that impede the management of patients with health issues [15]. Therefore, the aim of this study was to evaluate the knowledge, attitudes, and practices of patients with ESKD with regard to hemodialysis and its complications.

## Methods

### Study design and subjects

This cross-sectional study enrolled patients with uremia who were receiving hemodialysis at the Second Affiliated Hospital of Nanjing Medical University (Nanjing, China) between January 9, 2023, and January 16, 2023. The inclusion criteria were as follows: (1) receiving maintenance hemodialysis; (2) the study objective and informed consent for participation; and (3) being considered capable of filling out the questionnaire accurately. The exclusion criteria were: (1) mental disorder or incapable to accurately responding to the items in the questionnaire; and (2) receiving hemodialysis via a central venous catheter. The study was approved by the Medical Ethics Committee of the Second Affiliated Hospital of Nanjing Medical University and informed written consent was obtained from all the study participants.

### Design and distribution of the questionnaire

The questionnaire was designed by the study authors, and the first draft was then modified according to comments made by two experts in hemodialysis (a chief physician in the nephrology center and an attending physician in the nephrology department). The finalized questionnaire was administered to 64 patients undergoing maintenance hemodialysis as a pretest, and the pretest results indicated that the questionnaire had good reliability (a Cronbach’s α value of 0.720, suggesting good internal consistency) (Supplementary Tables S1-S3).

The final version of the questionnaire was in Chinese and contained four dimensions: demographic/clinical information, knowledge, attitude, and practice. The demographic/clinical characteristics collected by the questionnaire included gender, age, education level, area of residence, income, whether living alone, marital status, type of medical insurance, duration of hemodialysis, cause of uremia, type of vascular access, source of knowledge. The knowledge dimension consisted of 11 questions. The response questions were scored 1 point for a correct answer or 0 points for an incorrect or unclear answer. For response questions, 1 point was awarded for each correct choice, and 0 points were awarded for an incorrect choice or unclear answer. The total score for the knowledge dimension ranged from 0 to 31 points. The attitude dimension consisted of 8 questions, which were scored using a 5-point Likert scale (“strongly agree” = 5 points, “strongly disagree” = 1 point). The total score for the attitude dimension ranged from 8 to 40 points. The practice dimension contained 11 questions and was scored using a 5-point Likert scale depending on the option selected (“always”, “often”, “sometimes”, “occasionally”, or “never”). The total score for the practice dimension ranged from 11 to 55 points.

A paper version of the questionnaire was administered to each study participant while they were undergoing hemodialysis by a bedside nurse with more than 2 years of professional experience. The nurse conducted a face-to-face interview with the patient and filled in the questionnaire according to the responses, and the patient was then asked to check the contents of the completed questionnaire. Before the interview, the patient was informed about the purpose of the interview and asked to sign a consent form. During the interview, attention was paid to maintaining communication with the patient and avoiding interruptions, leading questions, and subjective evaluations.

### Statistical analysis

Stata 17.0 (Stata Corporation, College Station, TX, USA) was used for the analysis. Continuous data are expressed as the mean ± standard deviation, and categorical data are expressed as *n* (%). Normally-distributed continuous variables were compared between groups using Student’s t-test (two groups) or analysis of variance (three or more groups). Non-normally distributed continuous variables were compared using the Wilcoxon-Mann-Whitney test (two groups) or Kruskal-Wallis analysis of variance (three or more groups). Correlations between dimension scores were evaluated using Pearson correlation analysis. *P* < 0.05 was considered statistically significant.

## Results

### Demographic and clinical characteristics of the study participants

The final analysis included completed questionnaires from 493 patients (305 males, 61.87%) undergoing maintenance hemodialysis. The demographic and clinical characteristics of the study participants are summarized in Table 1. Approximately half of the respondents (250/493, 50.71%) were aged 45–65 years old, with only 88 participants (17.85%) aged < 45 years old. The majority of respondents were married (402/493, 81.54%), not living alone (399/493, 81.43%), and residing in an urban area (391/493, 79.31%). More than 60% of the participants were educated to middle school, high school, or secondary school level (309/493, 62.68%), with around one-fifth educated to college level or higher (102/493, 20.69%). The level of income was < 10,000 RMB/year for 186 participants (37.73%) and > 30,000 RMB/year for 170 respondents (34.48%). All but one of the patients (99.80%) had medical insurance. The main cause of uremia was metabolic disease (240/493, 48.78%). Most of the patients (303/493, 61.71%) had been receiving hemodialysis for more than 5 years, and the most common form of vascular access was arteriovenous fistula (400/493, 81.14%). The main methods by which the respondents obtained hemodialysis-related information included health promotion programs (437/493, 88.64%), public WeChat accounts and video channels (315/493, 63.89%), interactions with other patients (272/493, 55.17%) and websites (201/493, 40.77%).

**Table 1:** Baseline characteristics of the study participants

Characteristic	Value
Gender, *n* (%)	
Male	305 (61.87)
Female	188 (38.13)
Age (years), mean ± SD	57.58 ± 12.64
< 45 years-old, *n* (%)	88 (17.85)
45–65 years-old, *n* (%)	250 (50.71)
≥ 65 years-old, *n* (%)	155 (31.44)
Area of residence, *n* (%)	
Rural	55 (11.16)
Urban	391 (79.31)
Suburban	47 (9.53)
Education level, *n* (%)	
Below primary school	82 (16.63)
Middle/high/secondary school	309 (62.68)
College/bachelor’s degree or above	102 (20.69)
Income, *n* (%)	
< 10,000 RMB/year	186 (37.73)
10,000–20,000 RMB/year	87 (17.65)
20,000–30,000 RMB/year	50 (10.14)
> 30,000 RMB/year	170 (34.48)
Home living situation, *n* (%)	
Living alone	91 (18.57)
Not living alone	399 (81.43)
Marital status, *n* (%)	
Unmarried	47 (9.53)
Married	402 (81.54)
Other	44 (8.92)
Medical insurance, *n* (%)	492 (99.80)
Hemodialysis duration, *n* (%)	
< 1 year	42 (8.55)
1–2 years	49 (9.98)
2–5 years	97 (19.76)
> 5 years	303 (61.71)
Cause of uremia, *n* (%)	
Glomerulonephritis	104 (21.14)
Metabolic disease	240 (48.78)
Hereditary kidney disease	42 (8.54%
Other	106 (21.54)
Vascular access, *n* (%)	
Arteriovenous fistula	400 (81.14)
Arteriovenous graft	78 (15.82)
Brachial artery superficialization / direct radial artery puncture	15 (3.04)
Methods of learning, *n* (%)	
Health promotion programs	437 (88.64)
Public WeChat accounts and video channels	315 (63.89)
Websites	201 (40.77)
Patient-to-patient communication	272 (55.17)
Other	42 (8.52)

SD: standard deviation

### Knowledge score

The mean knowledge score was 19.33 ± 7.07 points (possible range, 0–31 points), suggesting that the surveyed patients had a moderate level of knowledge about the complications of hemodialysis. The proportion of respondents giving correct answers to each of the 11 questions in the knowledge dimension ranged from 24.34 to 98.17% (Table 2). More than half the respondents (60.65%) correctly defined the chronic kidney disease stage corresponding to uremia (item 1), but only 24.34% of the participants knew the best treatment for uremia (item 2). Most patients recognized edema (70.18%), loss of appetite/nausea/vomiting/diarrhea (68.15%) and tiredness/mental depression (66.33%) as clinical manifestations of uremia, but memory loss/insomnia (51.93%) and uremic fetor (46.45%) were recognized by fewer patients (item 6). More than half the respondents were aware of the potentially fatal complications of long-term hemodialysis (69.78%; item 3) and the most common cardiovascular complications of hemodialysis (56.19%; item 4). Among the acute complications of hemodialysis (item 7), symptomatic hypotension (73.83%) and hypertension during dialysis (69.57%) were recognized by most patients, but fewer were aware of dialysis disequilibrium syndrome (53.14%) or bleeding during dialysis (41.78%). Most patients knew that secondary hyperparathyroidism and renal osteodystrophy (67.14%) and cardiovascular system complications (64.71%) were chronic complications of hemodialysis (item 8), but fewer were aware of infection-related complications (52.13%), digestive system abnormalities (47.87%) and dialysis-associated amyloidosis (31.24%). Only around half of the patients (51.52%) knew that aneurysm was a common complication of arteriovenous fistula (item 9), whereas more respondents were aware of thrombosis (73.43%), infection (68.76%), and vascular stenosis (64.50%). Most of the participants correctly answered questions relating to the interdialytic weight gain limit (61.05%; item 5) and the dietary principles for patients on hemodialysis (79.92–98.17%; item 10). Finally, the proportion of correct responses to questions relating to clinical signs of complications ranged from 49.90% for ischemic stroke syndrome to 85.40% for diminished/inaudible arteriovenous fistula murmur on auscultation (item 11).

**Table 2:** Responses to the items in the knowledge dimension

Item	Correct response
What stage of chronic kidney disease is called uremia?	299 (60.65%)
Best treatment for uremia	120 (24.34%)
Long-term hemodialysis complications leading to death	344 (69.78%)
Most common cardiovascular complications of hemodialysis	277 (56.19%)
Control range for weight gain between dialysis sessions	301 (61.05%)
Clinical manifestations of uremia	
Tiredness and mental depression	327 (66.33%)
Loss of appetite, nausea, vomiting, diarrhea	336 (68.15%)
Lower extremity or generalized edema with occasional hydrothorax/ascites	346 (70.18%)
Uremic fetor	229 (46.45%)
Memory loss, insomnia	256 (51.93%)
Acute complications of hemodialysis	
Symptomatic hypotension	364 (73.83%)
Hypertension on dialysis	343 (69.57%)
Disequilibrium syndrome	262 (53.14%)
Bleeding during dialysis	206 (41.78%)
Chronic complications of hemodialysis	
Secondary hyperparathyroidism and renal osteodystrophy	331 (67.14%)
Dialysis-associated amyloidosis	154 (31.24%)
Digestive system abnormalities	236 (47.87%)
Infection-related complications	257 (52.13%)
Cardiovascular system complications	319 (64.71%)
Common complications of arteriovenous endovascular fistula	
Thrombosis	362 (73.43%)
Infection	339 (68.76%)
Vascular stenosis	318 (64.50%)
Aneurysm	254 (51.52%)
Dietary principles for patients on hemodialysis	
Low-salt diet	441 (89.45%)
Low-phosphorus diet	394 (79.92%)
High-potassium diet (incorrect option)	484 (98.17%)
High-quality protein diet	403 (81.74%)
Symptom recognition at the clinic	
Visual examination for infection or limb swelling	338 (68.56%)
Aneurysms that have ruptured or are at risk of rupture	287 (58.22%)
Ischemic steal syndrome (limb coldness, numbness or pain)	246 (49.90%)
Abnormal pulsation or vibration on palpation	375 (76.06%)
Diminished or inaudible internal fistula murmur on auscultation	421 (85.40%)

Subgroup analyses (Table 3) indicated that a higher knowledge score was associated with younger age (*P* < 0.001), a higher education level (*P* < 0.001), not living alone (*P* < 0.001), and glomerulonephritis as the cause of uremia (*P* < 0.001), whereas vascular access via implantation of a arteriovenous graft was associated with a lower knowledge score (*P* < 0.001). The knowledge score did not differ significantly between groups stratified according to gender, place of residence, income, marital status, or duration of hemodialysis (Table 3).

**Table 3:** Questionnaire dimension scores stratified according to demographic and clinical characteristics

Characteristic	Knowledge			Attitude			Practice		
	Mean	SD	*P*	Mean	SD	*P*	Mean	SD	*P*
Total score	19.33	7.07		28.77	3.58		43.57	6.53	
Gender			0.708			0.158			0.550
Male	19.33	7.45		28.53	3.60		43.31	6.73	
Female	19.35	6.42		29.17	3.53		43.99	6.18	
Age			< 0.001			0.141			0.346
< 45 years-old	22.40	7.18		28.84	4.01		43.10	7.30	
45–65 years-old	19.38	6.67		28.55	3.49		43.44	6.21	
≥ 65 years-old	17.53	7.07		28.10	3.48		44.04	6.58	
Area of residence			0.589			0.032			0.264
Rural	18.60	6.92		28.45	2.67		42.42	6.57	
Urban	19.46	6.81		28.95	3.68		43.73	6.51	
Suburban	19.19	9.17		27.68	3.59		43.60	6.65	
Education			< 0.001			0.276			0.334
Below primary school	16.09	7.69		28.57	3.15		43.22	7.79	
Middle/high/secondary school	19.17	6.75		28.70	3.58		43.35	6.30	
College/bachelor’s degree or above	22.43	6.25		29.17	3.93		44.51	6.06	
Income, RMB/year			0.079			0.458			0.159
< 10,000	18.39	7.52		28.88	3.58		43.15	7.26	
10,000–20,000	19.43	6.86		28.09	3.25		42.78	6.06	
20,000–30,000	19.04	6.66		28.74	3.29		45.50	5.53	
> 30,000	20.41	6.69		29.02	3.82		43.87	6.09	
Home living			< 0.001			0.576			0.072
Living alone	16.42	7.00		28.75	3.33		42.19	7.80	
Not living alone	19.99	6.92		28.80	3.64		43.90	6.18	
Marital status			0.264			0.598			0.073
Unmarried	20.09	8.71		28.30	3.71		42.15	7.77	
Married	19.35	6.94		28.84	3.59		43.85	6.39	
Other	18.43	6.39		28.66	3.41		42.55	6.17	
Hemodialysis duration, years			0.078			0.567			< 0.001
< 1	21.07	5.59		28.98	3.56		46.90	5.99	
1–2	19.86	7.96		29.10	2.69		43.12	5.79	
2–5	20.21	6.64		28.94	3.67		45.24	6.00	
> 5	18.73	7.17		28.67	3.68		42.70	6.63	
Cause of uremia			< 0.001			0.215			0.970
Glomerulonephritis	21.87	6.39		28.58	3.54		43.58	5.99	
Metabolic disease	18.94	6.93		29.13	3.57		43.58	6.50	
Hereditary kidney disease	16.86	6.31		28.69	3.69		43.69	7.81	
Other	18.65	7.70		28.15	3.55		43.58	5.99	
Vascular access			0.001			0.423			0.987
Arteriovenous fistula	19.85	7.08		28.74	3.61		43.52	6.68	
Arteriovenous graft	16.88	6.54		28.78	3.42		43.96	5.57	
Brachial artery superficialization / direct radial artery puncture	18.33	7.29		29.67	3.89		43.00	7.30	

SD: standard deviation

### Attitude score

**Fig. 1:**
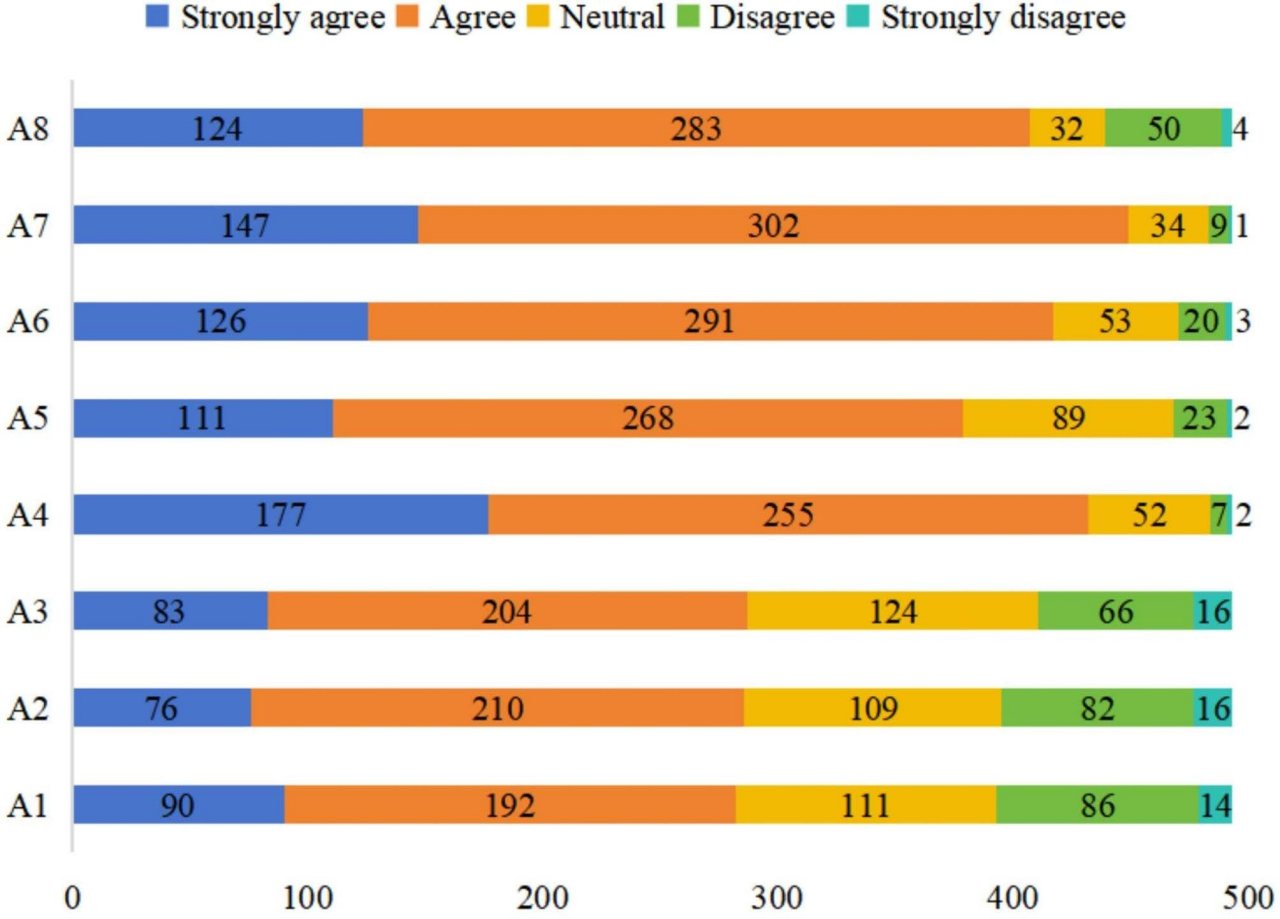
Responses to the items in the attitude dimension. **A1:** My illness has substantially affected my normal social interactions with family, friends, neighbors or other groups; **A2:** I am willing to have regular hemodialysis, which does not have too great effect on my life financially or psychologically; **A3:** Hemodialysis makes me feel a sense of helplessness about my life; **A4:** I would like to learn more about uremia and hemodialysis to prolong my hemodialysis; **A5:** Despite the need for hemodialysis, I have short-, medium- and/or long-term goals for my life; **A6:** I am confident regarding the treatment of uremia; **A7:** I should have a positive attitude and take action to improve my health status; **A8:** With regard to uremia, I believe that I can live a normal life as long as I adhere to the treatment

The average attitude score was 28.77 ± 3.58 (possible range, 8–40 points), indicating that the participants had only a moderately positive attitude toward hemodialysis. The distributions of the responses to the 8 questions in the attitude dimension are summarized in Fig. 1. The vast majority of respondents (> 75%) strongly agreed or agreed that they should have a positive attitude and take action to improve their clinical status (item 7) and that they have short-term, medium-term, and/or long-term life goals despite the need for hemodialysis (item 5). However, only 58.02% of respondents strongly agreed or agreed with the statement that they were willing to have regular hemodialysis and that doing so would not greatly affect them financially or psychologically (item 2). Furthermore, 58.22% of patients strongly agreed or agreed that hemodialysis gave them a feeling of helplessness (item 3), and 57.21% of patients strongly agreed or agreed that their illness has substantially affected their normal social interactions with their family, friends, neighbors or others (item 1). Notably, attitudes toward dealing with uremia were more positive: 87.62% of patients indicated that they would like to learn more about hemodialysis and uremia to prolong their lifespan (item 4), 84.59% of participants were confident in the treatment of uremia (item 6), and 82.55% of respondents agreed that they could live a normal life as long as they adhered to the treatment for uremia (item 8).

Although the attitude score varied significantly between subgroups stratified according to the place of residence (urban, suburban, or rural), the observed differences were small. Furthermore, the attitude score did not vary according to the other demographic and clinical characteristics (Table 3).

### Practice score

**Fig. 2:**
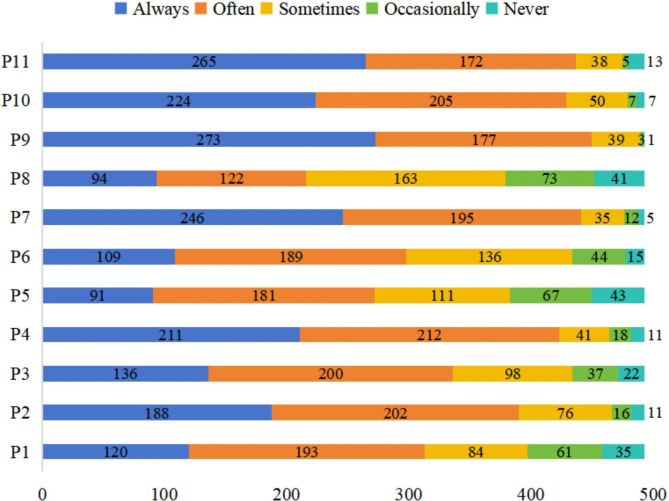
Responses to the items in the practice dimension. **P1:** I measure my blood pressure at home regularly; **P2:** After each blood test, I actively check the test results or ask the medical staff about my test results; **P3:** I deliberately choose foods that are in line with the dietary recommendations; **P4:** At home, I follow the nurse’s instructions to check whether the vibration or murmur of the arteriovenous fistula is normal; **P5:** I control my water intake so that my daily weight gain does not exceed 1 kg; **P6:** I learn about hemodialysis through various means; **P7:** I adjust the tourniquet after hemodialysis according to hemostasis requirements; **P8:** I am comfortable talking to health care professionals about my psychological distress; **P9:** I take my medication as directed by my healthcare provider; **P10:** At home, I inspect the puncture area for any redness or swelling; **P11:** I minimize movement of my arm during hemodialysis

The practice score for the respondents averaged 43.57 ± 6.53 points (possible range, 11–55 points). As shown in Fig. 2, more than 85% of the participants stated that they always or often followed the nurse’s instructions to check whether the vibration or murmur of the arteriovenous fistula was normal (85.80%; item 4), adjusted the tourniquet after hemodialysis according to hemostasis requirement (89.45%; item 7), took their medications as directed by their healthcare provider (91.28%; item 9), checked the puncture area for redness or swelling when at home (87.02%; item 10), and minimized movement of their arm during hemodialysis (88.64%; item 11). Additionally, 79.10% of the patients always or often made active efforts to check the results of their medical tests (item 2). However, fewer respondents always or often took their blood pressure regularly at home (63.49%; item 1), selected foods in line with dietary advice (68.16%; item 3), and controlled their water intake so that their daily weight gain did not exceed 1 kg (55.17%; item 5). Moreover, only 43.82% of the patients indicated that they were comfortable talking to healthcare professionals about psychological distress (item 8).

The practice score was highest for patients who had been undergoing hemodialysis for less than 1 year but lowest for those who had been receiving hemodialysis for more than 5 years (*P* < 0.001; Table 3). The practice score was comparable between groups stratified according to the other baseline characteristics (Table 3).

### Correlations between the knowledge, attitude, and practice scores

Correlation analysis did not identify a significant correlation between the knowledge score and attitude score (*r* = 0.028, *P* = 0.542). However, there was a positive correlation between the knowledge and practice scores (*r* = 0.220, *P* < 0.001) and between the attitude and practice scores (*r* = 0.453, *P* < 0.001).

## Discussion

Notable findings of this study were that patients with uremia undergoing hemodialysis in Nanjing (China) had moderate levels of knowledge, attitude and practice with regard to hemodialysis and its complications. Furthermore, the practice score was significantly positively correlated with both the knowledge and attitude scores, although there was no significant correlation between the knowledge and attitude scores. To our knowledge, this is the first survey evaluating the knowledge, attitudes, and practices of patients undergoing hemodialysis in China with regard to the complications of hemodialysis. Our survey results provide new insights that may help to guide the development and implementation of targeted interventions to improve the self-management skills of patients on maintenance hemodialysis.

The participants in this study had a mean knowledge score of 19.33 points, indicating that they had only a moderate level of knowledge about hemodialysis and its complications. Our finding that the level of knowledge regarding hemodialysis complications was not high is consistent with previous reports suggesting that patients undergoing hemodialysis have a suboptimal level of knowledge in other hemodialysis-related areas. For example, Ghannadi et al. reported that knowledge about self-management was low in patients with type 2 diabetes mellitus who were receiving hemodialysis (mean score of 59.90 ± 11.23 points out of a possible maximum of 100 points) [16]. Four different studies found that knowledge of dietary guidelines was suboptimal in 49.4% [17], 39.7% [18], 52.9% [19] and 83.3% [20] of patients receiving maintenance hemodialysis. A survey by Pessoa et al. showed that 97.7% of patients on hemodialysis had inadequate knowledge relating to the arteriovenous fistula [21], and a study by Iqbal et al. also identified deficiencies in knowledge regarding fistula care [22]. Thus, a general finding is that patients receiving maintenance hemodialysis have knowledge deficiencies in several areas relating to hemodialysis therapy and its complications.

Areas of deficient knowledge identified in the present study included the best treatment for uremia, memory loss/insomnia, and uremic fetor as manifestations of uremia, dialysis disequilibrium syndrome and bleeding during dialysis as acute complications of hemodialysis, infection-related complications, digestive system abnormalities, and dialysis-associated amyloidosis as chronic complications of hemodialysis, and aneurysm as a common complication of arteriovenous fistula. Previous research has indicated that education programs can improve the knowledge of patients on hemodialysis [23–25]. Therefore, we suggest that the implementation of educational interventions may help to improve patients’ knowledge of hemodialysis and its complications.

The subgroup analyses performed in this study revealed that a higher knowledge score was associated with a higher education level, which would be consistent with previous research examining knowledge of dietary guidance in patients receiving hemodialysis [17, 18]. A higher knowledge score was also observed in younger respondents in this study, which would agree with a previous finding that younger patients receiving hemodialysis had better dietary knowledge than older patients [26]. Our study also identified not living alone as a factor associated with a higher knowledge score. Previous studies suggested that social support from family members helps to increase knowledge but also improves adherence to hemodialysis [27, 28]. This study did not examine adherence, but it could be considered for future ones.

The mean attitude score of approximately 29 out of a maximum of 40 indicates that, overall, the participants in this study had a moderately positive attitude toward hemodialysis and its complications. In general, our findings are in broad agreement with those of previously published studies. Ghannadi et al. found that the majority of their patients on hemodialysis had an unfavorable attitude to self-management (a mean score of 44.27 ± 8.35 points out of a maximum score of 100 points) [16]. Furthermore, positive attitudes to dietary recommendations were observed in 40.0–74.3% of patients on hemodialysis [17–20]. Additionally, around 70% of patients receiving hemodialysis had an adequate attitude to arteriovenous fistula self-care [21], and similar results were reported in another study [22]. In the present study, more than 75% of respondents had positive attitudes toward taking action to improve their clinical status and having life goals despite the need for hemodialysis, and treatment of uremia. However, a substantial proportion of participants indicated that regular hemodialysis would substantially affect them financially or psychologically, that hemodialysis gave them a feeling of helplessness, and that their illness had affected their social interactions with other people. Previous research has indicated that maintenance hemodialysis can have negative effects on psychological well-being and social interactions [29, 30]. Furthermore, poor financial status was reported to have a negative effect on the physical and psychological well-being of patients on maintenance hemodialysis [31]. Interventions to address these issues may help to improve the well-being and quality of life of patients on maintenance hemodialysis.

The average practice score was 43.57 ± 6.53 points out of a possible maximum of 55 points, suggesting that there was room for improvement in the practices of the patients with regard to self-management and avoidance of complications. Ghannadi et al. reported a low practice score of 45.06 ± 12.87 points in their cohort of patients on hemodialysis, which the authors speculated may have been due to a low level of education and a poor attitude toward their condition [16]. Suboptimal practices regarding adherence to dietary recommendations were reported in 37.1–61.4% of patients receiving hemodialysis [17–19]. Furthermore, 97.7% of patients on hemodialysis were found to have inadequate arteriovenous fistula self-care practices [21]. The above studies and others [22]suggest that the practices of patients receiving maintenance hemodialysis can be inadequate despite a moderately positive attitude. Specific deficiencies in practice identified in our study included regular blood pressure monitoring at home, selection of foods in line with dietary advice, controlling water intake to maintain daily weight gain within 1 kg, and talking to healthcare professionals about psychological distress. Notably, the practice score was positively correlated with both the knowledge score and attitude score. The above findings imply that interventions to enhance knowledge and attitude might lead to improvements in practice among people receiving maintenance hemodialysis. This concept is supported by published data demonstrating that educational interventions can help to improve the practices of patients on hemodialysis and their adherence to treatment [32–34]. Additional studies are merited to explore the effects of educational interventions on the knowledge, attitudes and practices of patients receiving maintenance hemodialysis.

This study has some limitations. First, the sample size was not particularly large, so it is possible that the analysis may have had insufficient statistical power to detect some real differences between groups. Second, this was a single-center study, so the generalizability of the findings remains unknown. Third, the KAP questionnaire may have limitations regarding its ability to evaluate perceptions of hemodialysis and its complications. Fourth, this study did not assess whether education programs would enhance the questionnaire scores or clinical outcomes. Finally, patients with a central venous catheter were excluded. Central venous catheters accounted for 9.8% of the patients on dialysis at the study center. Their average age was 79 years old, and they were generally unwilling to cooperate with the survey, leading to an even smaller sample size. Considering the limited data collected on these patients, patients with central venous catheters were excluded.

## Conclusions

In conclusion, the findings of this study provide important insights into the knowledge, attitudes, and practices of patients with uremia receiving hemodialysis in Nanjing (China) regarding hemodialysis and its complications. We anticipate that the findings will facilitate the development and implementation of education programs to enhance self-management practices in patients receiving maintenance hemodialysis.

### Electronic supplementary material

Below is the link to the electronic supplementary material.

Supplementary Material 1

Supplementary Material 2

Supplementary Material 3

## Data Availability

The data presented in this study are available in the article.
