# Supplementary material for: Knowledge, attitude, and practice of patients receiving maintenance hemodialysis regarding hemodialysis and its complications: a single-center, cross-sectional study in Nanjing
Source: BMC Nephrol. 2023 Sep 20;24:275. doi: 10.1186/s12882-023-03320-0 (PMC10510168; doi:10.1186/s12882-023-03320-0)
Supplement: Supplementary file 1 — Supplementary Material 1 [file 12882_2023_3320_MOESM1_ESM.docx]

Table S1. Responses to the items in the knowledge dimension of pilot experiment.

| **Item** | **Correct response** |
| --- | --- |
| What stage of chronic kidney disease is called uremia? | 29 (45.31%) |
| Best treatment for uremia | 19 (29.69%) |
| Long-term hemodialysis complications leading to death | 13 (20.31%) |
| Most common cardiovascular complications of hemodialysis | 30 (46.88%) |
| Control range for weight gain between dialysis sessions | 46 (71.88%) |
| Clinical manifestations of uremia |  |
| Tiredness and mental depression | 49 (76.56%) |
| Loss of appetite, nausea, vomiting, diarrhea | 47 (73.44%) |
| Lower extremity or generalized edema with occasional hydrothorax/ascites | 35 (54.69%) |
| Uremic fetor | 30 (46.88%) |
| Memory loss, insomnia | 38 (59.38%) |
| Acute complications of hemodialysis |  |
| Symptomatic hypotension | 53 (82.81%) |
| Hypertension on dialysis | 41 (64.06%) |
| Disequilibrium syndrome | 42 (65.63%) |
| Bleeding during dialysis | 38 (59.38%) |
| Chronic complications of hemodialysis |  |
| Secondary hyperparathyroidism and renal osteodystrophy | 53 (82.81%) |
| Dialysis-associated amyloidosis | 26 (40.63%) |
| Digestive system abnormalities | 35 (54.69%) |
| Infection-related complications | 39 (60.94%) |
| Cardiovascular system complications | 43 (67.19%) |
| Common complications of arteriovenous fistula |  |
| Thrombosis | 54 (84.38%) |
| Infection | 47 (73.44%) |
| Vascular stenosis | 47 (73.44%) |
| Aneurysm | 34 (53.13%) |
| Dietary principles for patients on hemodialysis |  |
| Low-salt diet | 61 (95.31%) |
| Low-phosphorus diet | 49 (76.56%) |
| High-potassium diet (incorrect option) | 10 (15.63%) |
| High-quality protein diet | 51 (79.69%) |
| Symptom recognition at the clinic |  |
| Visual examination for infection or limb swelling | 54 (84.38%) |
| Aneurysms that have ruptured or are at risk of rupture | 50 (78.13%) |
| Ischemic steal syndrome (limb coldness, numbness or pain) | 34 (53.13%) |
| Abnormal pulsation or vibration on palpation | 52 (81.25%) |
| Diminished or inaudible fistula murmur on auscultation | 54 (84.38%) |
