# Supplementary material for: Knowledge, attitude, and practice of patients receiving maintenance hemodialysis regarding hemodialysis and its complications: a single-center, cross-sectional study in Nanjing
Source: BMC Nephrol. 2023 Sep 20;24:275. doi: 10.1186/s12882-023-03320-0 (PMC10510168; doi:10.1186/s12882-023-03320-0)
Supplement: Supplementary file 2 — Supplementary Material 2 [file 12882_2023_3320_MOESM2_ESM.docx]

Table S2. Responses to the items in the attitude dimension of pilot experiment.

|  | Strongly agree | Agree | Neutral | Disagree | Strongly disagree |
| --- | --- | --- | --- | --- | --- |
| My illness has substantially affected my normal social interactions with family, friends, neighbors or other groups | 13 (20.31%) | 21 (32.81%) | 13 (20.31%) | 12 (18.75%) | 5 (7.81%) |
| I am willing to have regular hemodialysis, which does not have too great effect on my life financially or psychologically | 13 (20.31%) | 23 (35.94%) | 10 (15.63%) | 16 (25.00%) | 2 (3.13%) |
| Hemodialysis makes me feel a sense of helplessness about my life | 12 (18.75%) | 24 (37.50%) | 15 (23.44%) | 12 (18.75%) | 1 (1.56%) |
| I would like to learn more about uremia and hemodialysis to prolong my hemodialysis | 25 (39.06%) | 31 (48.44%) | 6 (9.38%) | 2 (3.13%) | 0 (0.00%) |
| Despite the need for hemodialysis, I have short-, medium- and/or long-term goals for my life | 9 (14.06%) | 33 (51.56%) | 15 (23.44%) | 6 (9.38%) | 1 (1.56%) |
| I am confident regarding the treatment of uremia | 19 (29.69%) | 28 (43.75%) | 13 (20.31%) | 2 (3.13%) | 2 (3.13%) |
| I should have a positive attitude and take action to improve my health status | 20 (31.25%) | 31 (48.44%) | 10 (15.63%) | 2 (3.13%) | 1 (1.56%) |
| With regard to uremia, I believe that I can live a normal life as long as I adhere to the treatment | 15 (23.44%) | 33 (51.56%) | 7 (10.94%) | 9 (14.06%) | 0 (0.00%) |
