# Supplementary material for: Knowledge, attitude, and practice of patients receiving maintenance hemodialysis regarding hemodialysis and its complications: a single-center, cross-sectional study in Nanjing
Source: BMC Nephrol. 2023 Sep 20;24:275. doi: 10.1186/s12882-023-03320-0 (PMC10510168; doi:10.1186/s12882-023-03320-0)
Supplement: Supplementary file 3 — Supplementary Material 3 [file 12882_2023_3320_MOESM3_ESM.docx]

Table S3. Responses to the items in the practice dimension of pilot experiment.

|  | **Always** | **Often** | **Sometimes** | **Occasionally** | **Never** |
| --- | --- | --- | --- | --- | --- |
| I measure my blood pressure at home regularly | 21 (32.81%) | 18 (28.13%) | 13 (20.31%) | 7 (10.94%) | 5 (7.81%) |
| After each blood test, I actively check the test results or ask the medical staff about my test results | 36 (56.25%) | 17 (26.56%) | 3 (4.69%) | 5 (7.81%) | 3 (4.69%) |
| I deliberately choose foods that are in line with the dietary recommendations | 21 (32.81%) | 18 (28.13%) | 14 (21.88%) | 4 (6.25%) | 7 (10.94%) |
| At home, I follow the nurse’s instructions to check whether the vibration or murmur of the arteriovenous fistula is normal | 31 (48.44%) | 17 (26.56%) | 10 (15.63%) | 3 (4.69%) | 3 (4.69%) |
| I control my water intake so that my daily weight gain does not exceed 1 kg | 16 (25.00%) | 19 (29.69%) | 10 (15.63%) | 13 (20.31%) | 6 (9.38%) |
| I learn about hemodialysis through various means | 19 (29.69%) | 17 (26.56%) | 14 (21.88%) | 9 (14.06%) | 5 (7.81%) |
| I adjust the tourniquet after hemodialysis according to hemostasis requirements | 46 (71.88%) | 14 (21.88%) | 4 (6.25%) | 0 (0.00%) | 0 (0.00%) |
| I am comfortable talking to health care professionals about my psychological distress | 15 (23.44%) | 15 (23.44%) | 15 (23.44%) | 4 (6.25%) | 15 (23.44%) |
| I take my medication as directed by my healthcare provider | 45 (70.31%) | 16 (25.00%) | 3 (4.69%) | 0 (0.00%) | 0 (0.00%) |
| At home, I inspect the puncture area for any redness or swelling | 39 (60.94%) | 19 (29.69%) | 1 (1.56%) | 3 (4.69%) | 2 (3.13%) |
| I minimize movement of my arm during hemodialysis | 50 (78.13%) | 7 (10.94%) | 2 (3.13%) | 3 (4.69%) | 2 (3.13%) |
